# A Midland Hospital and the Commissioners

**Published:** 1912-07-06

**Authors:** Leonard Willox

**Affiliations:** National Health Insurance Commission (Eng.), Buckingham Gate, London, S.W.


### THE EDITOR'S LETTER-BOX.

### A Midland Hospital and the Commissioners.

The following correspondence, which has passed between the General Hospital, Nottingham, and the Insurance Commissioners, has been sent to us for publication:—

National Health Insurance Commission,
London, S.W., June 19, 1912.

Gentlemen,—Will you please inform me whether resident medical officers in hospitals will become compulsory contributors under the Insurance Act, and, if so, what will be the respective contributions of the employers and the employees?

I would point out that the medical staff in hospitals is composed of qualified and registered medical men or women who receive as remuneration, speaking generally, board, lodging and washing, and salaries ranging from £40 to £100 a year, although in many hospitals no salary at all is given.

They are appointed for a period of from six to twelve months in most cases, after which time they may become engaged elsewhere at rates of remuneration which may, or may not, be above the £160 limit.

It is provided in Section 51 that "Where the managers of any institution carried on for *charitable* or reformatory purposes prove that the persons who are *inmates* of and supported by the institution receive maintenance and medical attendance when sick, the Insurance Commissioners may grant a certificate of exemption to those managers, and where such a certificate of exemption is granted, any such inmates who are employed by the managers of the institution shall not, in respect of such employment, be deemed to be employed within the meaning of the Act."

The vast majority of hospitals *are* charitable institutions, and medical men engaged in them, when ill, receive free medical attendance.

Could they therefore be classified as "inmates" under the foregoing section?

Again, what is the position, as regards insurance, of graduates who, as students, are attending post-graduate courses at colleges and universities for a period of months, and who, while so occupied, are not in receipt of any salary or private income?

I shall feel obliged if you will supply me with definite information on the points stated. Thanking you in anticipation,

I am, Gentlemen, yours faithfully,
Leonard Willox (M.B., Ch.B.).

National Health Insurance Commission (Eng.),
Buckingham Gate, London, S.W.,
June 29, 1912.

